# Further Investigations in Regard to “the Saliva as a Natural Protection against Caries”

**Published:** 1903-06

**Authors:** William H. Potter


### Reviews of Dental Literature.

Further Investigations in regard to "The Saliva as a Natural Protection against Caries." By Dr. Michel, Würzburg.[1]

The author asserts that the substance called in German "Rhodan" plays an important part in the household of nature, and that in every oral cavity the greater the quantity of this substance present, the fewer micro-organisms. "Rhodan" is the acid radicle of sulphocyanic acid, and has the formula CNS.

The protective power of saliva is exerted in a purely mechanical way in that it washes away injurious substances from the teeth, and also by its alkalinity, and thirdly, by the antifermentative action of the sulphocyanide of potassium contained therein. As a result of analyses of the saliva, the writer claims that it is proved that where there is an increased amount of carbonates, phosphates, and calcium in the saliva there is less caries of the teeth than where the saliva contains less of those substances which establish its alkalinity. It is also established on good authority that persons suffering from neurasthenia and anæmia have no sulphocyanic acid in the saliva, and it is well known that their teeth are much affected by caries. The use of tobacco is said to produce an increase of sulphocyanic acid in the saliva, and the author holds that, as a matter of observation, the resisting quality of the teeth is increased by such use. There follows a table giving the results of the examination of six hundred and four samples of saliva. The

[1] Weitere Untersuchungen über "Der Speichel als natürlicher Schutz gegen Caries," von Dr. med. Michel in Würzburg. Deutsche Monatsschrift für Zahnheilkunde, 27. Dezember, 1902.

mouths whence these samples were taken were examined as to their percentage of carious teeth, as to their reaction, and as to the amount of sulphocyanic acid in the saliva.

This table shows that where caries is least, alkalinity of the saliva is pronounced and sulphocyanic acid is at its maximum; where caries is most pronounced, the alkalinity of the saliva is much lessened and in many cases an acid reaction is present, and the quantity of sulphocyanic acid is much diminished and in some cases entirely wanting.

It remains to establish the action of sulphocyanic acid upon bacterial growth. The author says that saliva containing this substance will not cure an already established infection, but it will make difficult the development of all infectious elements. The author seems to believe it possible by internal medication to increase the amount of sulphocyanic acid in the saliva, and thus heighten the resistance of the teeth towards caries.

WILLIAM H. POTTER.

